# Axillary dissection versus axillary observation for low risk, clinically node-negative invasive breast cancer: a systematic review and meta-analysis

**DOI:** 10.1007/s12282-021-01273-6

**Published:** 2021-07-09

**Authors:** Mahaveer S. Sangha, Rose Baker, Muneer Ahmed

**Affiliations:** 1 University College London, London, WC1E 6DE UK; 2 Emeritus of Statistics, University of Salford, Maxwell Building, The Crescent, Salford, M5 4WT UK; 3 Breast Surgical Oncology, Division of Surgical and Interventional Sciences, University College London. Royal Free Hospital, 9th Floor (East). Pond St, London, NW3 2QG UK

**Keywords:** Axillary dissection, Axillary clearance, Early breast cancer, Axillary lymph nodes

## Abstract

**Purpose:**

1. To systematically analyse studies comparing survival outcomes between axillary lymph-node dissection (ALND) and axilla observation (Obs), in women with low-risk, clinically node-negative breast cancer. 2. To consider results in the context of current axillary surgery de-escalation trials and studies.

**Methods:**

9 eligible studies were identified, 6 RCTs and 3 non-randomized studies (4236 women in total). Outcomes assessed: overall survival (OS) and disease-free survival (DFS). The logged (ln) hazard ratio (HR) was calculated and used as the statistic of interest. Data was grouped by follow-up.

**Results:**

Meta-analyses found no significant difference in OS at 5, 10 and 25-years follow-up (5-year ln HR = 0.08, 95% CI − 0.09, 0.25, 10-year ln HR =  0.33, 95% CI − 0.07, 0.72, 25-year ln HR = 0.00, 95% CI − 0.18, 0.19). ALND caused improvement in DFS at 5-years follow-up (ln HR = 0.16, 95% CI 0.03, 0.29), this was not demonstrated at 10 and 25-years follow-up (10-year ln HR = 0.07, 95% CI − 0.09, 0.23, 25-year ln HR = − 0.03, 95% CI − 0.21, 0.16). Studies supporting ALND for DFS at 5-years follow-up had greater relative chemotherapy use in the ALND cohort.

**Conclusion:**

ALND does not cause a significant improvement in OS in women with clinically node-negative breast cancer. ALND may improve DFS in the short term by tailoring a proportion of patients towards chemotherapy. Our evidence suggests that when the administration of systemic therapy is balanced between the two arms, axillary de-escalation studies will likely find no difference in OS or DFS.

**Supplementary Information:**

The online version contains supplementary material available at 10.1007/s12282-021-01273-6.

## Introduction

Lack of evidence-based demonstration of survival benefit in landmark trials such as NSABP B-04 [1] and ACOSOG Z0011 [2] have been pivotal in reducing the extent of surgery in breast cancer. Currently, the SOUND [3] trial aims to determine whether there is a therapeutic role in sentinel lymph node biopsy (SLNB) over observation alone in low-risk breast cancer with normal preoperative axillary imaging. However, studies pre-dating the SLNB era, which compared axillary lymph node dissection (ALND) to observation (Obs) in clinically node-negative women with invasive breast cancer, can shed light on the likely direction results, from axillary de-escalation trials, will take. A previous review by Sanghani et al*.* [4] conducted a three-way comparison between Obs, ALND and axillary radiotherapy and reported no differences in survival. The review was limited to a follow-up of 5-years, and only two (of four) studies compared axillary ALND to Obs. In this review, studies pre-dating SLNB and comparing ALND to Obs only are comprehensively assessed and a meta-analysis is conducted to examine the difference in long-term outcomes between ALND and Obs in women with low-risk, clinically node-negative breast cancer. Furthermore, the relevance of results to current practice and future research on axillary de-escalation is considered.

## Methods

### Study design

The study was conducted in accordance with the Preferred Reporting Items of Systematic Reviews and Meta-Analyses (PRISMA) guidance.

### Search process and study selection

**Fig. 1:**
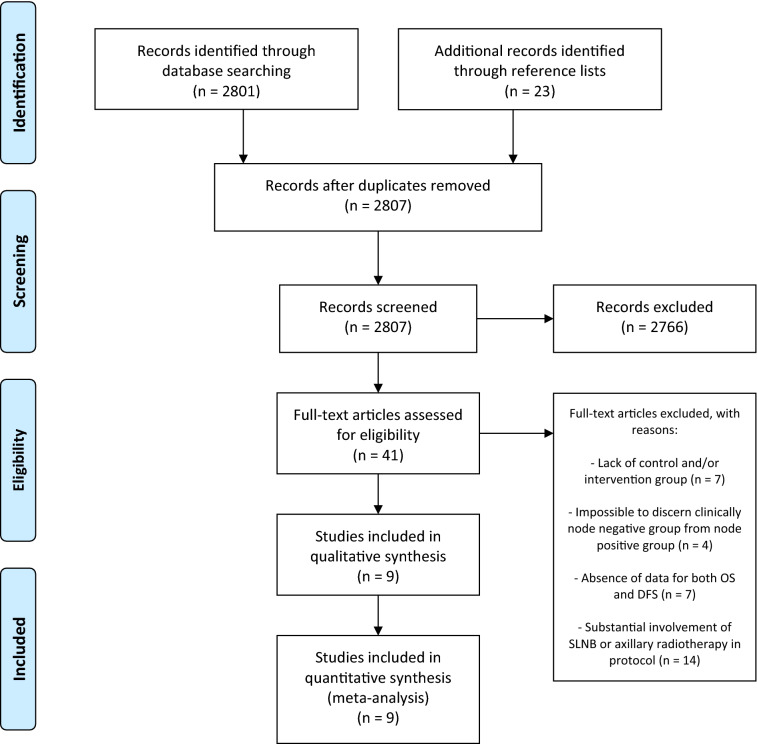
Results at each stage of systematic study selection

A systematic literature search was conducted using PubMed/MEDLINE and Cochrane databases. Search terms included: breast cancer, node negative, axillary dissection, clearance and radical mastectomy. The search, and title and abstract screening were conducted by one author (M.S.), full article screening was conducted independently by two authors (M.S. and M.A.), disagreements in study selection were resolved through discussion. Results of each stage are illustrated in Fig. 1. Reference lists of screened articles were also reviewed. Date of the last search: 15th April 2021.

### Inclusion and exclusion criteria

All studies comparing long-term outcomes between ALND and Obs of the axilla in women with clinically node-negative invasive breast cancer were eligible. Studies must have reported at least one measure of long-term outcome that could be extracted from text or figures. Measures were pre-specified as overall survival (OS) and disease-free survival (DFS) due to relative consistency in reporting. Studies must have been published in the English language. Studies not fulfilling the inclusion criteria were excluded, no restrictions were placed on study design or year of publication.

### Bias assessment

**Fig. 2:**
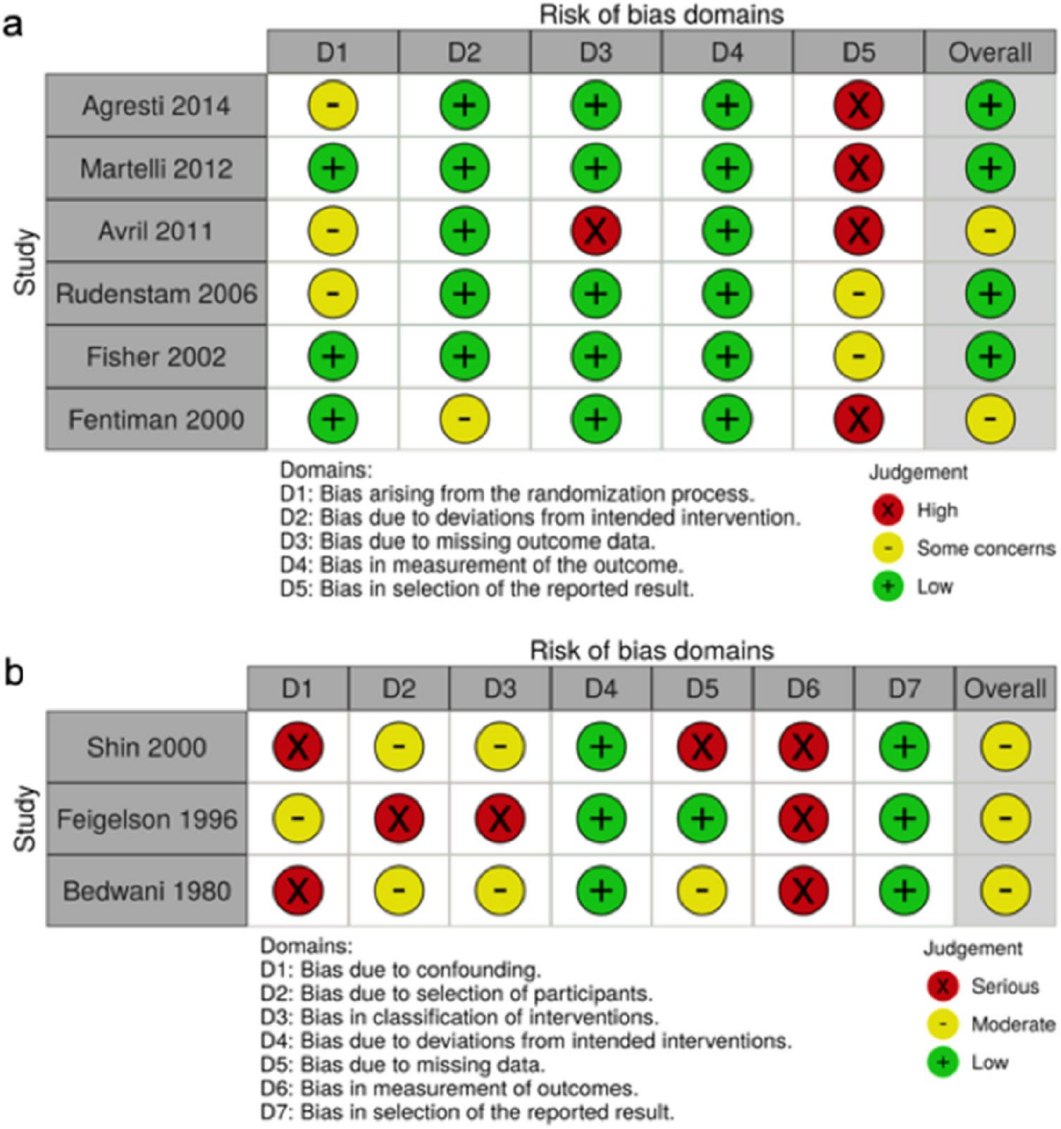
Results of bias analyses. **a** Summary table of results from RoB 2 bias analysis of RCTs**. b** Summary of results from ROBINS-I bias analysis of RCTs

Bias assessment was carried out by two authors (M.S. and M.A.) independently, then reviewed jointly for discrepancies and re-assessment. Bias was assessed in accordance with the Cochrane Handbook [5]. For randomised control trials (RCTs), the ‘revised Cochrane risk of bias tool for randomized trials’ (RoB 2) [6] was used. For non-randomised studies, the ‘Risk Of Bias In Non-randomized Studies—of Interventions’ (ROBINS-I) tool [7] was used. Overall assessment of bias is presented in Fig. 2.

### Data collection and statistical analysis

Study data was extracted by one author (M.S.) and reassessed by a second (M.A.) Where available, the following variables (presented in Table 1) were collated: total sample size, control/intervention sample size, mean/median age, follow-up period, OS and DFS at each follow-up interval and corresponding hazard ratios (HR), axillary recurrence, the proportion of ALND group with involved lymph nodes, proportion of cohort with T1 staged disease, proportion with oestrogen receptor (ER) positive disease and proportion of cohort receiving chemotherapy, radiotherapy and endocrine therapy. Where OS and DFS were available from survival curves only, the PlotDigitizer [8] software was used to extract data.

**Table 1:** Study characteristics

Study	Study Design	Intervention	Control	Recruitment Period	Follow Up	Total Participants	Mean/Median Age	T1 %	ER+ % Intervention vs. Control*	Proportion total mastectomy % vs. all subtotal resection %		Proportion Menopausal	Received Chemotherapy %	Received Radiotherapy %	Received Endocrine Therapy %	Specific Concerns Over Bias
Agresti 2014 [20]	RCT	Axillary Dissection	No Axillary Intervention	1998–2003	10	517	﻿52.6	98.8	80.9 vs. 81.6	Intervention: 0 vs. 100 Control: 0 vs. 100		–	35.5	100	35.5	Randomization: assigned unmasked and based on enrolment
Martelli 2012 [21]	RCT	Axillary Dissection	No Axillary Intervention	1987–1992	15	219	70	92.7	85.4 vs. 89.1	Intervention: 0 vs. 100 Control: 0 vs. 100		100	0	100	100	–
Avril 2011 [17]	RCT	Axillary Dissection	No Axillary Intervention	1995–2005	5	607	62.6	–	85 vs. 79	Intervention: 5 v 95 Control: 3 vs. 97		100	2	97	91	Recruitment: halted recruitment early Randomization: intraoperative Censoring: obligatory at 5–years despite evidence for impact on results
Rudenstam 2006 [22]	RCT	Axillary Dissection	No Axillary Intervention	1993–2002	5	473	74	57	76 vs. 84	Intervention: 45 vs. 55 Control: 44 vs. 56		100	0	32	100	Randomization: pseudo random sequencing
Fisher 2002 [1]	RCT	Radical Mastectomy	Mastectomy without Axillary Intervention	1971–1974	25	1079	56.4	–	–	Intervention: 100 vs. 0 Control: 100 vs. 0		70	–	–	–	–
Shin 2000 [18]	Non-Randomised Trial	Partial Axillary Dissection	No Axillary Intervention	1996-2000	5	252	66	50	69 vs. 62	Intervention: 37 vs. 63 Control: 0 vs. 100		17.9	3	60	84	Sample size: small Obs cohort
Fentiman 2000 [25]	RCT	Halstead Mastectomy	No Axillary Intervention	1971–1975	10	255	58	27.2	–	Intervention: 100 vs. 0 Control: 0 vs. 100		–	–	–	–	–
Feigelson 1996 [23]	Retrospective Study	Axillary Dissection	No Axillary Intervention	1983–1994	5	78	74.3	100	–	Intervention: NA Control: NA		100	0	28.2	33	Sample size: small Obs and ALND cohort
Bedwani 1980 [19]	Cohort Study	Any Axillary Surgery	No Axillary Intervention	1978–Undisclosed	5	1108	56.9	–	–	Intervention: 100 vs. 0 Control: 82 vs. 18		–	–	–	–	Analysis: no method to control for confounding

ER+: patients with oestrogen receptor positive breast cancer only; NA: not available; *collated oestrogen only and oestrogen and progesterone receptor positive breast cancer

Data were analysed by authors R.B. and M.S. An odds ratio (OR), as seen in other reviews, was not deemed suitable as DFS/OS (cumulative survival data) cannot be reliably converted into an OR and no studies reported crude survival data. There was an additional issue where few studies reported hazard ratios or standard errors for OS/DFS at each interval of follow-up. Instead an approach suggested by Moodie et al*.* [9], for meta-analyses of survival data where HR and SE are not reported, was considered appropriate and viable after discussion with statistician colleagues. Data processing was carried out by author R.B. using Fortran90 [10]–[12]. Code is presented in supplementary materials and utilised data for: OS, DFS and number at risk. A million simulations were ran to calculate the HR and SE for each study. From this, values for logged (ln) HR, ln SE and 95% confidence intervals (CI) for each study were acquired and used as the statistics of interest for meta-analyses, results are presented in Table 2. Meta-analyses were grouped according to follow-up interval (5, 10 and 25-years) and were conducted using RevMan 5.4.1 [13]. A random/fixed-effects model was used depending on the presence/absence of heterogeneity, respectively [14]. Heterogeneity was evaluated using the Chi^2^ test [15]. Egger’s Funnel Plot was used to assess for publication bias.

**Table 2:** Study results after processing

Study	Patients in ALND Group	Patients in Obs Group	5-Year Follow-Up Ln OS HR	10-Year Follow-Up Ln OS HR	25-Year Follow-Up Ln OS HR	5-Year Follow-Up Ln DFS HR	10-Year Follow-Up Ln DFS HR	25-Year Follow-Up Ln DFS HR	Axillary Recurrence in ALND Group %	Axillary Recurrence in Obs Group %	Cases with Involved LN in ALND Group %
Agresti 2014 [20]	272	245	0.625 ± 0.5917	0.1268 ± 0.1381	–	− 0.1001 ± 0.3302	0.1194 ± 0.1339	–	10y: 0	10y: 9	28.7
Martelli 2012 [21]	109	110	− 0.520 ± 0.4934	–	–	–	–	–	15y: 0	15y: 6	23
Avril 2011 [17]	310	297	0.2540 ± 0.1527	–	–	0.2809 ± 0.1467	–	–	5y: 0	5y: 1.3	14
Rudenstam 2006 [22]	234	239	− 0.0423 ± 0.1693	–	–	− 0.0698 ± 0.1510	–	–	5y: 1	5y: 3	28
Fisher 2002 [1]	362	365	0.0278 ± 0.1476	0.0948 ± 0.1172	0.0045 ± 0.0932	0.1204 ± 0.1168	0.0421 ± 0.1046	− 0.0266 ± 0.0945	10y: 1.4, 25y: 4	10y: 1.1, 25y: 6	–
Shin 2000 [18]	207	45	0.1412 ± 0.8615	–	–	0.5599 ± 0.7715	–	–	5y: 0	5y: 4.4	25
Fentiman 2000 [25]	133	122	–	0.9481 ± 0.2560	–	–	–	–	–	–	–
Feigelson 1996 [23]	64	14	− 0.2017 ± 0.7786	–	–	–	–	–	–	–	–
Bedwani 1980 [19]	926	182	–	–	–	0.3359 ± 0.1390	–	–	–	–	–

Ln: logged; HR: hazard ratio; ALND: axillary dissection cohort; Obs: axillary observation only cohort; OS: overall survival; DFS: disease free survival; y: years

## Results

### Selected studies

2824 studies were identified. The final review found nine suitable studies: six RCTs, one non-randomised control trial, one retrospective study and one cohort study (Table 1).

### Study characteristics

Study characteristics are presented in Table 1. 2617 patients were assigned to ALND and 1619 to Obs. OS was defined as the interval from randomization to the last point of follow-up or death from all causes in all studies. DFS was defined as the interval from randomization to death, first recurrence of disease in the breast, axilla or elsewhere, or last follow-up in all studies.

### Risk of bias within and across studies

Bias assessment results are presented in Fig. 2 with individual comments in Table 1. Of the six RCTs, four included power analyses [16, 19]–[21], none achieved sufficient numbers. There were concerns over randomization techniques used by three RCTs. Blinding was not feasible in any study. Details on withdrawn participants were given in all studies and did not impact results. Concerns over censorship and missing outcome data were present in one study [16]. One study ended patient recruitment early [16] and one extended the recruitment window [20]. All studies had some concerns over selective reporting of result statistics.

Of the three non-randomized studies, two included small sample sizes [17, 22]. One cohort study [18] lacked an appropriate method to control for confounding and the study design allowed for selection bias. Concerns over bias due to measurement outcomes were present in all three studies.

Funnel plots were symmetrical and suggested no publication bias.

### Results of individual studies

The RCT by Agresti et al*.* [18] compared ALND to Obs in women aged 30–65. No significant difference in OS (HR = 1.09, 95% confidence interval (CI) 0.59–2.00, *p* = 0.783) and DFS (HR = 1.04, CI 0.56–1.94, *p* = 0.898) at 10-years follow-up.

The RCT by Martelli et al*.* [20] compared ALND to Obs in post-menopausal women aged 65–80. No significant difference in hazard of death (HR = 1.18, CI 0.73–1.92), breast cancer-related mortality (HR = 0.721, CI 0.27–1.89, *p* =  0.51) and distant metastases (HR = 1.572, CI 0.70–3.50, *p* =   0.27) was found at 15-years follow-up.

**Fig. 3:**
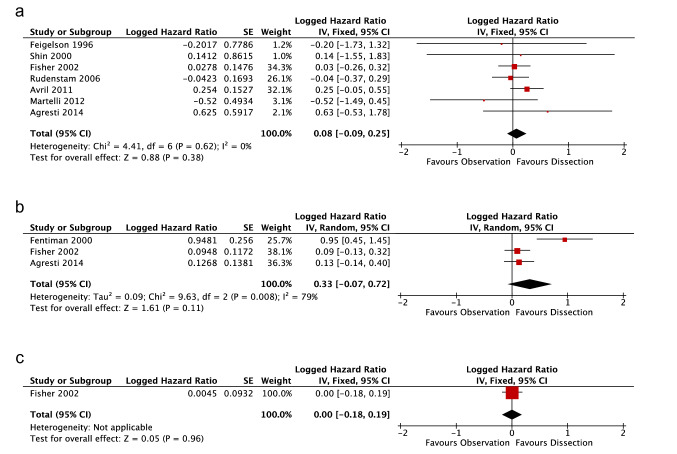
Meta-analysis and Forrest Plot for Overall Survival. **a** 5-year follow-up** b** 10-year follow-up **c** 25-year follow-up

**Fig. 4:**
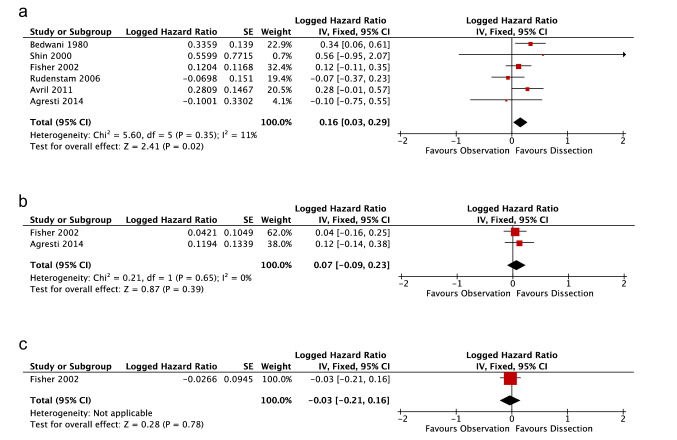
Meta-analysis and Forrest Plot for Disease-Free Survival. **a** 5-year follow-up** b** 10-year follow-up **c** 25-year follow-up

The RCT by Avril et al*.* [16] compared ALND to Obs in post-menopausal women aged > 50. Significant difference in OS (HR: 3.07, 90%C I 1.40–6.70, *p*  = 1) and DFS (HR = 2.26, 90% CI 1.32–3.86, *p* = not reported), was found at 5-years follow-up. Our statistical analyses, using the study’s data, emulated these findings but did not demonstrate significance in either OS or DFS (Figs. 3a and 4a).

The RCT by Rudenstam et al*.* [21] compared ALND to Obs in post-menopausal women. No significant difference in OS (HR = 1.05, CI 0.76–1.46, *p* = 0.77) and DFS (1.06, CI 0.79–1.42, *p* = 0.69) was found at 5-years follow-up.

The RCT by Fisher et al*.* [1] compared radical mastectomy (RM) to mastectomy without axillary radiation (M) in pre-, peri- and post-menopausal women. No significant difference between RM and M was demonstrated at 25-years (HR = 1.03, CI 0.87–1.23, *p* = 0.72 and HR = 1.10, CI 0.89–1.35, *p* =   0.39 for OS and DFS, respectively), 10-years (OS: RM vs. M: 58 ± 2.6 vs. 54 ± 2.7, and DFS: RM vs. M: 47 ± 2.6 vs. 42 ± 2.6) and 5-years follow-up (OS: RM vs. M: 75 ± 2.3 vs. 74 ± 2.3 and DFS: RM vs. M: 60 ± 2.5 vs. 56 ± 2.5).

The RCT by Fentiman et al*.* [23] compared Halstead Mastectomy (HM) to wide local excision (WLE) in women aged > 50. The study reported worse survival outcome after a mean follow-up of 9-years (HM vs. WLE: 82% vs. 60%) and increased loco-regional relapse (8% vs. 30%, respectively).

The non-randomised trial by Shin et al*.* [17] compared ALND to Obs in women aged 24–90. No significant difference in OS (ALND vs. Obs: 98 vs. 98) and DFS (96 vs. 93) was found at 5-years follow-up.

The retrospective study by Feigelson et al*.* [22] compared breast surgery with ALND (AS) to lumpectomy only (LO) in menopausal women aged 70–95. No significant difference in OS (AS vs. LO: 82.8 vs. 85.7) was found. Of note, non-axillary surgical approaches were not balanced between arms.

The cohort study by Bedwani et al*.* [18] compared radical surgeries (RS) to non-radical surgery (NRS) in pre-, intra- and post-menopausal women. They found no significant difference in DFS (RS vs. NRS: 62.7 vs. 63.2) at 5-years follow-up.

### Logged Overall Survival Hazard Ratio Meta-Analysis

At 5-years follow-up there were seven viable studies. Studies showed no heterogeneity (*I*^2^ = 0%, *χ*^2^ = 4.41, *p* = 0.62), thus a fixed-effect analysis was used. Meta-analysis for ln OS HR showed no significant difference (0.08, CI -0.09, 0.25; Fig. 3a) between ALND and Obs. Removal of non-randomised studies resulted in no change in ln HR for OS at 5-years follow-up (0.08, CI:-0.09, 0.25).

At 10-years, three studies remained. There was significant heterogeneity between studies (*I*^2^ = 79%, *χ*^2^ = 9.63, *p* = 0.008; Fig. 3b), thus a random-effects analysis was used. Meta-analysis showed no significant difference in OS (0.33, CI − 0.07, 0.72). The study by Fentiman et al*.,* which strongly opposed observation, used comparatively low-dose adjunct therapy, which may have contributed to heterogeneity. Removal of this study results in a ln HR that is in keeping with 5- and 25-years follow-up and does not show heterogeneity (0.11, CI -0.07 to 0.28, *I*^2^ = 0%, χ^2^ = 0.03, *p* = 0.86).

Only Fisher et al*.* produced data for 25-years follow-up [1], equivalence in OS ln HR (0.00, CI − 0.18, 0.19) was demonstrated.

It appears that data favours equivalence the longer the follow-up interval.

### Logged disease-free survival hazard ratio meta-analysis

At 5-years follow-up there were six viable studies. Studies showed little heterogeneity (*I*^2^ = 11%, *χ*^2^ = 5.60, *p* = 0.35), thus a fixed-effect analysis was used. Meta-analysis for DFS ln HR showed significance and favoured dissection (0.16, CI 0.03, 0.29; Fig. 4a). Removal of non-randomised studies results in DFS favouring axillary dissection at 5-years follow-up but no longer significant (0.10, CI − 0.05, 0.25).

At 10-years, two studies remained. There was no heterogeneity between studies (*I*^2^ = 0%, *χ*^2^ = 0.21, *p* = 0.65; Fig. 4b), thus a fixed-effect analysis was used. Meta-analysis demonstrated no significant difference between Obs and ALND (0.07, CI − 0.09, 0.23).

Only Fisher et al*.* produced data for 25-years follow-up, which demonstrated no significant difference in DFS (− 0.03, CI − 0.21, 0.16).

As with OS, data from later follow-up intervals favours equivalence more so than short-term.

## Discussion

Meta-analyses demonstrate that the lack of survival advantage from ALND presented in individual studies was not due to a lack of power and was not limited by the age of the cohort.

No significant difference in OS at 5, 10 and 25-years follow-up was identified between ALND and Obs. DFS was significantly greater in the ALND cohort at 5-years follow-up, but became non-significant at 10- and 25-years follow-up and shifted towards equivalence.

Our findings are supported by a previous study by Sanghani et al. [4] which compared axillary radiotherapy, dissection and observation. Authors found no improvement in OS when comparing dissection to radiotherapy (1 study) and observation (2) or when comparing radiotherapy to observation (1). Conversely, the study found no significant difference in DFS at 5-years, but was limited to a small study selection, comparisons between multiple interventions/protocol and an inappropriate statistical technique [9].

Additionally, we report that non-significant difference in survival is sustained over longer follow-up, confirming that treatment after recurrence benefits survival in the Obs cohort and early recurrence in the Obs cohort does not lead to increased reduction in long-term survival. Moreover, studies analysed in Sanghani et al*.*’s review were limited to an older patient cohort, our findings suggest that ALND does not improve survival irrespective of age.

### Disease-free survival

Despite initial significant improvement in DFS in the ALND cohort, it does not appear to influence OS. Systemic adjuvant treatment, specifically chemotherapy, may explain this result as it has been shown to improve survival [24]. We posit that ALND identifies the presence of axillary metastases and tailors the sub-group towards adjuvant treatment that more effectively reduces the rate of recurrence. In the Obs cohort, patients who have a relapse of disease undergo ALND and are treated with a similar protocol and thus overall survival is not impacted in the long-term.

When examining studies individually, four [16, 17, 19, 21] showed variation in adjuvant therapy between ALND and Obs groups and two [1, 18] did not disclose adjuvant therapy use. Three studies utilised adjuvant chemotherapy in the treatment protocol, all reported greater usage in the ALND cohort (Obs vs. ALND: Shin: 3% vs 29%; Avril: 2% vs 8%; Agresti: 35.5% vs 51.5%). Two studies (Shin et al*.* and Avril et al*.*) reported greater DFS in ALND over Obs, with a large relative difference in chemotherapy usage between the two cohorts. However, ln HR was not significant in either. The Agresti study had the smallest relative difference in chemotherapy and DFS is marginally in favour of Obs, though not significant.

These findings support the notion that chemotherapy may be responsible for improving early DFS by eliminating metastatic disease that is conducive to early recurrence, and that ALND is able to select for disease that is responsive to chemotherapy and which would otherwise recur in a 5-year interval.

This may also be true for SLNB. Studies such as the Z0011 trial [2] and more recently a study by Shigematsu et al*.* [25] had participants undergo SLNB before assignment to Obs or ALND. Both reported no difference in the proportion of participants receiving adjuvant chemotherapy and, as expected, no difference in 5-year DFS.

### Axillary recurrence

Although ALND appears to select for a disease that benefits from chemotherapy, it cannot identify, with a high degree of sensitivity, the small cohort of women who have metastases that lead to recurrences.

Previous analyses [26] have highlighted increased axillary recurrence in the Obs cohort and its lack of association with the number of patients with histologically involved nodes in the ALND cohort [4]. Although not formally assessed, data presented within this study (Table 2) leans towards the support of this. Our results suggest axillary recurrence in the Obs cohort does not correlate with the proportion of patients in the ALND group with histologically node-positive disease, nor with a significant difference in DFS beyond short-term follow-up.

This finding supports a hypothesis initially proposed by Veronesi et al*.* [27] that postulated the presence of indolent metastases that are unlikely to lead to disease recurrence and more aggressive metastases that do lead to recurrence.

Genotypic differences in metastases may explain the presence of a small cohort of women who have aggressive microscopic, metastatic foci that lead to occult recurrence rather than remaining indolent. In this group, ALND or SLNB may be necessary to reduce disease progression. But, as the recurrence cohort is small, this will expose many women to unnecessary surgery and a greater risk of comorbidities.

Genomic assays present an alternative and non-invasive solution that can identify loci which confer a recurrence risk in initially clinically node-negative women. The efficacy of genomic assays for this purpose should be investigated in future studies.

### In the context of modern trials

Current ongoing trials are examining the extent to which ALND should be omitted, our review of pre-SLNB studies can provide some insight into the expected results of these.

The SENOMAC trial [28] is randomising patients with T1-3 primary breast tumours and up to two axillary macrometastases to either SLNB only or SLNB with dissection. Parallels with our study can be drawn, for instance authors argue that previous trials omitted key groups such as those who underwent mastectomy and therefore ALND cannot be confidently ruled out. All three studies (Shin et al*.,* Fentiman et al*.* and Bedwani et al*.*), analysed in this review that involved mastectomy, supported ALND at 5- and 10-years follow-up respectively, suggesting patients undergoing mastectomy (who likely have higher grade disease) benefit from dissection. It is important to note that the (neo)adjuvant therapy regimen, which we argue is critical in equating survival between cohorts, in these three studies differs from the regimens of the modern era. Similarly, SLNB may guide no ALND patients towards specific and more intense therapy than if the axillary status remained unknown. Therefore no difference in survival is an equally plausible result from the SENOMAC study.

The SERC trial [29] is examining the value of ALND in patients with breast cancer of higher risk features. Currently reported underpowered results suggest that chemotherapy use significantly reduces the presence of disease in sentinel nodes and more so when given prior to dissection. Considering our results suggest few histologically node-positive patients undergo disease recurrence, reducing the burden further with chemotherapy prior to ALND/SLNB implies that even SLNB may not be needed when neoadjuvant chemotherapy is administered in women with breast cancer of select characteristics.

Our findings can be extrapolated to suggest that trials such as TAXIS [30] and Alliance A011202 [31], which are examining the effect of radiotherapy (RT) on the undissected and dissected axilla, will not identify improved survival outcomes. It is plausible that loco-regional recurrence may be reduced by RT in these studies. Therefore, the short-term improvement in DFS caused by ALND may be minimised as the undissected group will also be receiving targeted axillary (radio)therapy which prior studies have established is equivocal to dissection in the low-risk cohort [32]. This is supported by that fact that our result of significant improvement in DFS at 5-years follow-up differs from similar reviews [33, 34] that included axillary RT with no surgical intervention and found no significant difference in DFS over a similar interval.

In the context of the SOUND [3] and INSEMA [35] trials, which are comparing SLNB to Obs in low-risk breast cancer, our findings suggest that Obs will unlikely be inferior to SLNB, especially considering the eligible cohort is of a lower risk than patients recruited in studies analysed by this review.

### Qualitative analysis of variables

All studies, excluding Fentiman et al*.*, Feigelson et al*.*, Bedwani et al*.* and Shin et al*.*, provided evidence to support balanced patient assignment to surgical procedure. Studies with unbalanced surgical therapy assignment had more extensive non-axillary surgery in the ALND group compared to Obs. There was support for ALND improving OS at 10-years follow-up by Fentiman et al. Additionally, there was support for ALND improving DFS at 5-years follow-up by Bedwani et al*.* and Shin et al. It is unlikely these studies skewed results towards favouring dissection as meta-analysis excluding these sub-optimal studies does not alter results dramatically. When all other parameters are controlled, it is unlikely the primary breast surgery type exacerbates any effect ALND has on OS or DFS.

No study reported a significant difference in the proportion of patients with positive oestrogen/progesterone receptors between study arms. Two studies reported greater endocrine therapy use in the Obs cohort (Shin: 84% vs 70%; Avril: 91% vs 66%) and showed increased DFS in ALND (non-significant ln HR). One study reported greater endocrine therapy usage in the ALND cohort (Agresti: 41% vs 56%) and favoured Obs for DFS (non-significant). Endocrine therapy is unlikely to truly detriment DFS as other reports suggest otherwise[36]. Chemotherapy preceded endocrine therapy in the Agresti study and was used only in patients with poor characteristics; it is likely chemotherapy in the Obs cohort positively influenced DFS rather than endocrine therapy correlating with DFS decline in the ALND cohort. Radiotherapy usage was similar between ALND and Obs cohorts and is thus unlikely to influence DFS at 5-years follow-up.

Perioperative therapy changed between 1996 and 2014. Increasing efficacy of non-surgical therapeutics may tend recent studies towards equivalence and historic studies towards ALND. This was not demonstrated by our results which instead suggested an association with unbalanced chemotherapy assignment between arms. Instead recurrence rates may have reduced over time as perioperative treatment protocols shifted. Due to the lack of completeness of reported data, this could not be assessed.

## Limitations and future direction

Our study was limited by the lack of declared values for HR (of OS and DFS) and standard error, and variation in follow-up periods. This was mitigated through appropriate statistical techniques.

Of the studies assessed, a single study by Avril et al*.*, which suggested ALND improved both OS and DFS, was attenuated when adjusted based on our statistical analysis. This is not concerning, however, as authors reported a HR < 1.6 would support equivalence and the lower confidence intervals of declared data for OS and DFS are below this value (1.40 and 1.32, respectively). Our statistical analyses mirror these findings, showing that Avril et al*.*’s data is in favour of OS and DFS at 5-years follow-up but the results are not significant. Of further note, Avril et al*.* also reported a trend towards equivalence from uncensored data in all parameters except DFS, which supports our analysis.

A single study recorded survival at 25-years follow-up, more data is required to confirm equivalent survival over extended follow-up.

Future studies should assess the risk of comorbidities from SLNB when compared to axillary Obs only, and the value of genomic assessment compared to SLNB in stratifying patients into those who would benefit from further axillary therapy and those who would not.

Qualitative analysis was conducted on pre-specified data. Comprehensive and quantitative subgroup analysis was not possible due to limited access to raw data, such an analysis may yield information on the variation of treatment effect in subgroups and should be attempted in future studies.

Non-randomised studies were included in this study with the aim to comprehensively analyse all data available and generate representative findings. Unfortunately, this increased risk of confounding bias. The extent was minimised by strict non-randomised study bias assessment by two authors. Moreover, the undue effect from non-randomised studies is unlikely to be substantial, as these studies were weighted less, and analysis demonstrated equivocal findings upon their removal.

## Conclusion

The results of this study indicate long-term equivalence in OS between ALND and Obs in all women with early-stage low-risk breast cancer, however, some improvement in DFS is seen in the ALND cohort in the short-term. It is unlikely difference in OS or DFS will be identified in axillary de-escalation studies of clinically node-negative breast cancer when the administration of systemic therapy is balanced between the two arms. However, the value of ALND, and possibly SLNB, may be in its ability to tailor a proportion of patients towards chemotherapy and thus DFS improvement, but this does not translate to OS benefit as relapses are treated by further interventions.

## Supplementary Information

Below is the link to the electronic supplementary material.

Supplementary file1 (DOCX 22 kb)

## Data Availability

The datasets generated during and/or analysed during the current study are available from the corresponding author on reasonable request.

## Code availability

The code generated in the current study is available within the supplementary information files.
