# Supplementary material for: Axillary dissection versus axillary observation for low risk, clinically node-negative invasive breast cancer: a systematic review and meta-analysis
Source: Breast Cancer. 2021 Jul 9;28(6):1212–24. doi: 10.1007/s12282-021-01273-6 (PMC8514376; doi:10.1007/s12282-021-01273-6)
Supplement: Supplementary file 1 — Supplementary file1 (DOCX 22 kb) [file 12282_2021_1273_MOESM1_ESM.docx]

**Supplementary Materials for**

**Title:** Axillary Dissection versus Axillary Observation for Low Risk, Clinically Node-Negative Invasive Breast Cancer: A Systematic Review and Meta-Analysis

**Authors:** Mahaveer S Sangha^1^, Rose Baker^2^, Muneer Ahmed^3^

**Affiliations:**

^1^ Medical Student, University College London, London WC1E 6DE, United Kingdom. ORCID iD: 0000-0002-8839-4811

^2^ Emeritus Professor (School of Business), University of Salford, Maxwell Building, The Crescent, Salford M5 4WT, United Kingdom. ORCID iD: 0000-0003-3555-3425

^3^ Consultant in Breast Surgical Oncology, Division of Surgical and Interventional Sciences. University College London. Royal Free Hospital, 9th Floor (East). Pond St. London NW3 2QG, United Kingdom. ORCID iD: 0000-0002-4504-1354

**Corresponding Author (pre-publication enquiries):**

Mr Mahaveer S Sangha, University College London, London WC1E 6DE

E-mail: [mahaveer.sangha.16@ucl.ac.uk](mailto:mahaveer.sangha.16@ucl.ac.uk)

**This file includes:**

Supplementary Code for Materials and Methods

**Materials and Methods**

Methodology

Moodie *et al.* [10] address the problem of conducting meta-analyses using survival data when the hazard ratio and its standard error are not disclosed. With sample size *N* and an exponential survival time *S* = exp(−λ*t*) (constant hazard of death or disease recurrence) the logged hazard ratio λ_1_/λ_2_ is:

$$\hat{\theta} = ln(-ln(S_{1})) - ln(-ln(S_{2})).$$

When a constant hazard is not assumed, *θ*  is the logged ratio of average hazard functions over the time period. The standard error on $\hat{\theta}$ can be approximated using the delta-method and is given in the study by Moodie *et al*. It can be found more accurately by simulation: using the probabilities *S*_1_, *S*_2_ of survival for each group, samples can be simulated and their standard error found. When this is done, the result is close to the delta-method approximation, but on average slightly larger. A problem occurs with taking logarithms when the simulated *S* = 0 or *S* = 1, and in this case half a patient, or *N* −1/2 patients, are assumed to have survived to make *θ* finite. Moodie *et al.* also give the standard error of $\hat{\theta}$ assuming that *θ* = 0, the null hypothesis of equal hazards. This would be used for tests where the hazards are equal, as opposed to computing a confidence interval on *θ*.

In practice, the two errors are very similar numerically for these data. The normality of $\hat{\theta}$ is assumed by the methodology, and the simulation showed typically very low skewness and kurtosis, confirming this necessary fact. Moodie *et al*. discuss the problem of loss to follow-up. Clearly, if just after starting a study, half the patients vanished without a trace, the effective sample size would be only half of the nominal size *N*. This would inflate the standard error and change $\hat{\theta}$. This problem has been ignored here, and does not arise if ‘survival’ refers to patients who are known to be disease-free or at least alive, and those lost to follow-up are counted as diseased or dead.

A Fortran program was written to compute $\hat{\theta}$ and the various standard errors, using a million simulations.

Example of Input Data

The code below is an example of the input data used to generate logged (Ln) hazard ratio (HR) and standard error (SE) from data available in each study. The first and third column of integers represent the number at risk at the beginning of the study for the observation only (Obs) cohort and axillary dissection (AxD) cohort respectively. The second and fourth columns represent the number at risk after 5-years follow up. The first line represents values from overall survival (OS) analysis and the second line represents values from disease free survival (DFS) analysis.

Agresti

245 235 272 266

245 227 272 250

Program to Generate Logged Hazard Ratio and Standard Error

The source code below was used to generate and calculate the Ln HR and SE of each study at each year of follow-up. The code can be compiled using the NAG® Fortran Compiler or any other suitable compiler [13]. Commentary is given by author R.B.

module stuff

implicit double precision (a-h, o-z), integer (kind=3)(i-n)

save

integer, parameter :: mstate = 633, mseed = 1

! when copying to do random no. generation, add igenid=1

integer :: igenid, lseed, lstate, subid

integer :: seed(mseed), state(mstate)

end module stuff

program temp

use stuff

implicit double precision (a-h, o-z), integer (kind=3)(i-n)

double precision, parameter :: zero = 0.d+00, half = 0.5d+00, one = 1.d+00, two = 2.d+00, three = 3.d+00, four = 4.d+00, &

five = 5.d+00, pi = 3.14159265358979323846, epsilon = 1.d-08, eps = 1.d-03

character (80) :: infile, outfile, title(20), tname(2)

character (160) :: heading

!!!!!!!!!!!!!!!!!!!!!!!!!!!!!!!!!!!!!!!!!!!!!!!!!!!!!!!!!!!!!!!!!!!!!!!!

! !

! This program reads a datafile of survival data !

! and computes logged hazard ratio and standard error. !

! The standard error is computed by the delta method approximation, !

! and also using the Monte-Carlo simulation !

! which gives a more exact result. !

! The NAG library is used for generating random numbers, but can be !

! replaced by the fortran 90 intrinsic random_number(). !

!

!!!!!!!!!!!!!!!!!!!!!!!!!!!!!!!!!!!!!!!!!!!!!!!!!!!!!!!!!!!!!!!!!!!!!!!

integer :: n(2), ngone(2)

real *8 :: s(2), en(2), slog(2), prob(2), ssim(2), slogsim(2)

integer :: init(20, 2, 2), later(20, 2, 2)

tname(1) = 'Overall survival'

tname(2) = 'Disease Free Survival'

igenid = 1

nsims = 10000 !increase to 1 million to use in anger

ensims = dble(nsims)

infile = 'gethr5.dat'

open (9, file=infile, status='old', action='read', err=2)

outfile = 'gethr.lis'

open (10, file=outfile, status='replace', action='write', err=3)

open (11, file='gethr.ls1', status='replace', action='write', err=3)

call init_rand

read (9, '(a)', end=1) heading

write (10, '(a)') heading

do inum = 1, 100

read (9, '(a)', end=1) title(inum)

read (9, *, end=1)(init(inum,k,1), later(inum,k,1), k=1, 2)

read (9, *, end=1)(init(inum,k,2), later(inum,k,2), k=1, 2)

end do

1 ntodo = inum - 1

do itype = 1, 2 !OS and DFS

write (*, 110) itype

write (10, 100) tname(itype)

100 format (1x/a)

110 format ('processing type', i3)

nproc = 0

do ido = 1, ntodo

do k = 1, 2

n(k) = init(ido, k, itype)

if (later(ido,k,itype)==-1) exit !omit if missing value

ngone(k) = n(k) - later(ido, k, itype)

en(k) = dble(n(k))

s(k) = dble(n(k)-ngone(k))/en(k)

slog(k) = log(s(k))

prob(k) = s(k)

end do

if (later(ido,1,itype)==-1 .or. later(ido,2,itype)==-1) cycle !omit if missing value

theta = log(-slog(1)) - log(-slog(2)) !log hazard 1 -log hazard 2...+ve means gp 1 is worse

var = (one-s(1))/(s(1)*en(1)*slog(1)**2) + (one-s(2))/(s(2)*en(2)*slog(2)**2)

se = sqrt(var)

! now find se. under H0 that hazards are same

sav = (s(1)*en(1)+s(2)*en(2))/(en(1)+en(2))

var0 = (en(1)+en(2))**2*(one-sav)/(en(1)*en(2)*(en(1)+en(2)-one)*sav*log(sav)**2)

se0 = sqrt(var0)

write (10, 120) title(ido)

120 format (a)

write (10, 130) n(1), s(1), n(2), s(2)

130 format ('initial no. no diss.', i5, ' S', f12.4, ' initial no. diss.', i5, ' S', f12.4)

write (10, 140) theta, se, se0

140 format ('logged HR', f12.4, ' se', f12.4, ' se under H0', f12.4)

! get better se from simulation

thetasum = zero

thetasq = zero

cubed = zero

quad = zero

do i = 1, nsims

ngone = 0

! generate new sample

do k = 1, 2

do j = 1, n(k)

a = randu()

if (a>prob(k)) ngone(k) = ngone(k) + 1

end do

if (ngone(k)==0) then

ssim(k) = (en(k)-half)/en(k)

else if (ngone(k)==n(k)) then

ssim(k) = half/en(k)

else

ssim(k) = dble(n(k)-ngone(k))/en(k)

end if

slogsim(k) = log(ssim(k))

end do

thetanew = log(-slogsim(1)) - log(-slogsim(2))

thetasum = thetasum + thetanew

thetasq = thetasq + thetanew**2

cubed = cubed + thetanew**3

quad = quad + thetanew**4

end do

thetanew = thetasum/ensims

varnew = (thetasq-ensims*thetanew**2)/(ensims-one)

senew = sqrt(varnew)

sq = thetasq/ensims

cubed = cubed/ensims

quad = quad/ensims

skew = cubed - three*thetanew*sq + two*thetanew**3

skew = skew/senew**3

xkurt = quad - four*thetanew*cubed + 6.d+00*thetanew**2*sq - three*thetanew**4

xkurt = xkurt/varnew**2 - three

write (10, 150) nsims, senew, skew, xkurt

150 format ('se from', i8, ' simulations is', f12.4, ' skewness', f12.4, ' kurtosis', f12.4)

write (11, 160) theta, se, senew, se0

160 format (4g16.6)

nproc = nproc + 1

end do

write (*, 170) nproc

170 format (i5, ' studies processed')

write (11, '(1x)')

end do

stop 'done'

close (9)

close (10)

stop 'done'

2 write (*, '(''Unable to open input file '',a)') infile(:len_trim(infile))

stop 'failed'

3 write (*, '(''Unable to open output file '',a)') outfile(:len_trim(outfile))

stop 'failed'

end program temp

subroutine init_rand

use stuff

implicit double precision (a-h, o-z), integer (kind=3)(i-n)

!!!!!!!!!!!!!!!!!!!!!!!!!!!!!!!!!!!!!!!!!!!!!!!!!!!!!!!!!!!!!!!!!!!!!!!!

! !

! This routine initialises the NAG random-number generators. !

!

! !

!!!!!!!!!!!!!!!!!!!!!!!!!!!!!!!!!!!!!!!!!!!!!!!!!!!!!!!!!!!!!!!!!!!!!!!!

! Initialize the seed

seed(1) = 1762543

! IGENID and SUBID identify the base generator

subid = 1

! Initialize the generator to a repeatable sequence

lstate = mstate

lseed = mseed

ifail = 1

call g05kff(igenid, subid, seed, lseed, state, lstate, ifail)

end subroutine init_rand

double precision function randu()

use stuff

implicit double precision (a-h, o-z), integer (kind=3)(i-n)

!!!!!!!!!!!!!!!!!!!!!!!!!!!!!!!!!!!!!!!!!!!!!!!!!!!!!!!!!!!!!!!!!!!!!!!!

! !

! Returns a single random number. !

! Note, this is not the RANDU pseudorandom number generator. !

!

! !

!!!!!!!!!!!!!!!!!!!!!!!!!!!!!!!!!!!!!!!!!!!!!!!!!!!!!!!!!!!!!!!!!!!!!!!

real *8 :: ranvec(1)

ifail = 1

n = 1

call g05saf(n, state, ranvec, ifail)

randu = ranvec(1)

end function randu

double precision function gaussran()

!!!!!!!!!!!!!!!!!!!!!!!!!!!!!!!!!!!!!!!!!!!!!!!!!!!!!!!!!!!!!!!!!!!!!!!!

! !

! This routine returns a standard normal random deviate. !

! It uses Leva's algorithm, cited in !

! Numerical Recipes 3rd ed., page 369. [11] !

!

! !

!!!!!!!!!!!!!!!!!!!!!!!!!!!!!!!!!!!!!!!!!!!!!!!!!!!!!!!!!!!!!!!!!!!!!!!

real *8 :: u, v, x, y, q, randu

do

u = randu()

v = 1.7156*(randu()-0.5)

x = u - 0.449871

y = abs(v) + 0.386595

q = x**2 + y*(0.19600*y-0.25472*x)

if (q<0.27597 .or. (q<.27846 .and. v**2<-4.*log(u)*u**2)) exit

end do

gaussran = v/u

end function gaussran

Example of Output Data

The code below is an example of output data from the Fortran programme. The first section reports the OS Ln HR in the fifth line and the SE in the sixth line. The same is true for DFS in the second section of code.

Overall survival

Agresti

initial no. no diss. 245 S 0.9592 initial no. diss. 272 S 0.9779

logged HR 0.6250 se 0.5164 se under H0 0.5012

se from 1000000 simulations is 0.5917 skewness 0.4337 kurtosis 1.2438

Disease Free Survival

Agresti

initial no. no diss. 245 S 0.9265 initial no. diss. 272 S 0.9191

logged HR -0.1001 se 0.3179 se under H0 0.3171

se from 1000000 simulations is 0.3302 skewness -0.0745 kurtosis 0.2924

Data Processing

After acquisition of data from all relevant studies, meta-analysis was conducted using RevMan 5.4.1 [14]. A random/fixed-effects model was used depending on the presence/absence of heterogeneity [15]. Heterogeneity was evaluated using the Chi^2^ test [16]. Egger’s Funnel Plot was used to assess for publication bias.
